# The outer membrane secretin PilQ from *Neisseria meningitidis* binds DNA

**DOI:** 10.1099/mic.0.2006/004200-0

**Published:** 2007-05

**Authors:** Reza Assalkhou, Seetha Balasingham, Richard F. Collins, Stephan A. Frye, Tonje Davidsen, Afsaneh V. Benam, Magnar Bjørås, Jeremy P. Derrick, Tone Tønjum

**Affiliations:** 1 Centre for Molecular Biology and Neuroscience and Institute of Microbiology, University of Oslo, Oslo, Norway; 2 Centre for Molecular Biology and Neuroscience and Institute of Microbiology, Rikshospitalet-Radiumhospitalet Medical Centre, Oslo, Norway; 3 Faculty of Life Sciences, The University of Manchester, Manchester Interdisciplinary Biocentre, 131 Princess Street, Manchester M1 7DN, UK

## Abstract

*Neisseria meningitidis* is naturally competent for transformation throughout its growth cycle. Transformation in neisserial species is coupled to the expression of type IV pili, which are present on the cell surface as bundled filamentous appendages, and are assembled, extruded and retracted by the pilus biogenesis components. During the initial phase of the transformation process, binding and uptake of DNA takes place with entry through a presumed outer-membrane channel into the periplasm. This study showed that DNA associates only weakly with purified pili, but binds significantly to the PilQ complex isolated directly from meningococcal membranes. By assessing the DNA-binding activity of the native complex PilQ, as well as recombinant truncated PilQ monomers, it was shown that the N-terminal region of PilQ is involved in the interaction with DNA. It was evident that the binding of ssDNA to PilQ had a higher affinity than the binding of dsDNA. The binding of DNA to PilQ did not, however, depend on the presence of the neisserial DNA-uptake sequence. It is suggested that transforming DNA is introduced into the cell through the outer-membrane channel formed by the PilQ complex, and that DNA uptake occurs by non-specific introduction of DNA coupled to pilus retraction, followed by presentation to DNA-binding component(s), including PilQ.

## INTRODUCTION

*Neisseria meningitidis* (the meningococcus) is constitutively competent for transformation throughout its growth cycle. Natural transformation is a process unique to bacteria and involves active uptake of exogenous DNA from the environment, which can result in the acquisition of new genetic information in a heritable form, and is particularly important for genetic exchange and diversity (Chen & Dubnau, 2004). Transformation in Gram-negative bacteria differs from that in Gram-positive bacteria in that DNA has to pass through an additional layer, the outer membrane. Transformation is coupled to the expression of type IV pili in a number of Gram-negative bacteria, including *N. meningitidis* (Frøholm *et al.*, 1973), *Neisseria gonorrhoeae* (Mathis & Scocca, 1984; Swanson *et al.*, 1971), *Eikenella corrodens* (Tønjum *et al.*, 1985), *Moraxella nonliquefaciens* (Bøvre *et al.*, 1970; Bøvre, 1964), *Legionella pneumophila* (Stone & Kwaik, 1999), *Pseudomonas stutzeri* (Lorenz & Wackernagel, 1990) and *Thermus thermophilus* (Friedrich *et al.*, 2001). Type IV pili are long fibrous structures emanating from the bacterial surface, and as multifunctional organelles, they are involved in a variety of bacterial processes. In addition to their role in competence, the type IV pili of many species also play a role in adherence (Swanson *et al.*, 1971), twitching motility (Bradley, 1974; Mattick, 2002), biofilm formation (O'Toole & Kolter, 1998) and bacteriophage infection (Bradley, 1974). Twitching motility is driven by pilus retraction.

Efficient neisserial transformation further requires the presence of the frequently occurring 10 bp long signature sequence 5′-GCCGTCTGAA-3′, termed the DNA uptake sequence (DUS), in the transforming DNA (Goodman & Scocca, 1988). Homologous recombination of the incoming DNA with the chromosome is mediated by RecA (Koomey & Falkow, 1987), and is partially dependent upon other recombination components such as the RecBCD complex (Mehr & Seifert, 1998) and RecN (Skaar *et al.*, 2002). Current knowledge on the *N. gonorrhoeae* transformation process has recently been reviewed by Hamilton and Dillard (2006).

Neisserial competence for transformation is dependent on the expression of pilus biogenesis components (Carbonnelle *et al.*, 2006; Tønjum & Koomey, 1997) and several pilus-related components, including the minor pilin ComP (Wolfgang *et al.*, 1999; Aas *et al.*, 2002). Among the pilus biogenesis components are secretins, which belong to a large family of bacterial complexes associated with translocation of single proteins and macromolecules across the outer membrane. A subset of this family, termed PilQ proteins, is required for type IV pilus extrusion and retraction in *N. meningitidis*, *N. gonorrhoeae* and *Pseudomonas aeruginosa* (Drake & Koomey, 1995; Tønjum *et al.*, 1995). Neisserial PilQ null mutants are not piliated and are non-competent for natural transformation (Drake & Koomey, 1995; Tønjum *et al.*, 1998). Meningococcal PilQ is unique among secretins because of its abundance in the outer membrane and its N-terminally located polymorphic region containing small basic repeat (SBR) elements. We have previously shown that the native PilQ complex from meningococcal outer membranes adopts a cage-like structure of ∼900 kDa in total mass, consisting of 12 identical subunits (Collins *et al.*, 2001, 2003, 2004). Furthermore, we have provided evidence that PilQ and the pilus fibre interact, identified the region of PilQ that is involved in the PilQ–pilus interaction, and shown that this interaction induces a conformational change in the structure of the PilQ oligomer. These findings indicate that the PilQ complex is the channel through which its substrate, the moving pilus fibre (polymerized PilE), is directed to the bacterial surface (Collins *et al.*, 2005).

Transformation of neisserial DNA is unequivocally coupled to pilus expression, although the molecular basis for this correlation has not been elucidated. Mutations that affect the expression of type IV pili in *N. meningitidis* and *N. gonorrhoeae* greatly reduce the amount of DNA taken up during transformation (Koomey, 1998; Tønjum *et al.*, 1998; Tønjum & Koomey, 1997; Aas *et al.*, 2002). It is not clear, however, whether this phenomenon is solely due to the lack of pili per se, or to the direct involvement of pilus biogenesis components themselves in introducing DNA into the meningococcal cell. On the other hand, gonococcal pilin variants that do not express observable assembled pili, but express pilin subunits, can be competent for transformation to some extent (Long *et al.*, 2003). Due to its strong preference for DUS-containing DNA in transformation, the neisserial cell is able to distinguish between self DNA (containing DUS) and non-self DNA (lacking DUS). The DNA-binding events during transformation can thus be classified into two types: specific binding mediated by a putative DUS-specific receptor, and non-specific affinity mediated by non-specific DNA-binding component(s) that are required to support the entry of DNA into the cell. Among the components contributing to non-specific DNA binding during transformation, the periplasmatic protein ComE has been identified (Chen & Gotschlich, 2001).

In *P. aeruginosa*, evidence for direct binding of DNA to purified type IV pili has been obtained (van Schaik *et al.*, 2005), although this bacterial species is found to be non-competent for transformation. Here, we have addressed the question whether of the neisserial pilus itself and/or its corresponding secretin PilQ exhibit DNA-binding activity. The DNA-binding behaviour of purified pili and PilQ from meningococcal outer membranes was assessed by DNA band-shift, solution and solid-phase overlay assays, as well as by electron microscopy. We showed that purified pili from *N. meningitidis* and *P. aeruginosa* exhibit weak DNA binding, while the PilQ complex binds DNA to a significant extent. The PilQ-mediated binding of DNA was also assessed with regard to the structural location of the PilQ DNA-binding site, DUS specificity, and the relative preference for binding of ssDNA versus dsDNA. This work is important for the understanding of how DNA is introduced into the neisserial cell during the early phase of natural transformation.

## METHODS

### Bacterial strains and media.

The bacterial strains used in this study were *N. meningitidis* M1080 (Frasch & Chapman, 1972), M1080-Δ*pilQ* and TT31-221 (Tønjum *et al.*, 1998), *P. aeruginosa* PAK (Bradley, 1974), and *Escherichia coli* ER2566 (New England Biolabs). Strain TT31-221 contains chimeric PilQ, in which the N-terminal SBR region is replaced by the corresponding but non-homologous region in *P. aeruginosa* PilQ. Meningococcal and *E. coli* strains were grown as previously described (Tønjum *et al.*, 1995). Meningococcal strain M1080 was employed for PilQ complex purification. *P. aeruginosa* strain PAK was grown on Luria–Bertani (LB) or lactose medium. The M1080-Δ*pilQ* mutant has a transposon insertion in the *pilQ* start codon (Tønjum *et al.*, 1998).

### Bioinformatics analysis and prediction of secondary structure and electrostatic charge.

The deduced amino acid sequence of the *pilQ* of strain M1080 (GenBank accession no. AJ564200) was searched for the presence of recognized DNA-binding motifs and the electrostatic charge was calculated by using the charge program from the emboss package (Rice *et al.*, 2000).

### Cloning of the full-length and partial *pilQ*.

All standard methods of DNA manipulation were performed as previously described (Sambrook *et al.*, 1989; Tønjum *et al.*, 1995). The partial *pilQ* gene was PCR-amplified from genomic M1080 DNA (Frye *et al.*, 2006). For the generation of recombinant PilQ with an N-terminal His-tag, the vector pQE30 was used (Qiagen), and for the C-terminal His-tag, the pET28b(+) vector (Novagen) was used. The 5′ part of *pilQ* was amplified by using primers Q*Bam*HI and Q*Eco*RI, generating plasmid pPilQ-1. The central portion of *pilQ* was amplified with the primers SF21 and SF22, resulting in plasmid pPilQ-3. The 3′ part of *pilQ* was amplified with the primers SF20 and SF19 and subcloned into pET28b(+), resulting in plasmid pPilQ-4 (Table 1).

### Recombinant PilQ and MutY expression and purification.

For recombinant PilQ and MutY production, the plasmids were transformed into *E. coli* ER2566 (New England Biolabs), overexpressed, and the recombinant proteins were purified to homogeneity. For this purpose, cells were grown at 37 °C in LB broth until they reached OD_600_ 0.5, then protein overexpression was induced with 1 mM IPTG. Cells were grown for another 4 h, harvested by centrifugation and lysed by means of a French Press (Thermo Electron) at 20 000 p.s.i. (138 MPa). The His-tagged proteins were purified by immobilized metal affinity chromatography with nickel–nitrilotriacetic acid agarose (Qiagen), under native or denaturing conditions as recommended by the manufacturer. Protein concentrations were determined by Bradford analysis (Bradford, 1976), and the purity was determined by SDS-PAGE and Coomassie blue staining. Protein specificity was confirmed by immunoblotting using a polyclonal rabbit antiserum raised against the purified PilQ complex.

### Purification of native PilQ complex.

Purification of the PilQ complex from meningococcal outer membranes was performed as previously described (Collins *et al.*, 2001, 2003, 2004).

### Purification of type IV pilus fibres.

Type IV pili were purified from the *N. meningitidis* and *P. aeruginosa* cell surface using ammonium sulphate precipitation of a shearing fraction (Brinton *et al.*, 1978). Briefly, bacterial cells were vortexed for 1 min in 0.15 M ethanolamine buffer (pH 10.5), and cellular debris was removed by centrifugation. Pilus fibres were precipitated at room temperature for 30 min by addition of one-tenth volume ammonium sulphate-saturated 0.15 M ethanolamine buffer, and collected by centrifugation. Pili were subsequently washed twice with 50 mM Tris-buffered saline, pH 7.5, and treated with DNase (Sigma), prior to DNA-binding experiments to ensure that they were not naturally saturated with DNA.

### Rabbit immunization and antibody production.

Rabbit polyclonal antibodies were raised against the native PilQ complex, purified recombinant His-tagged PilQ fragments and purified pili, as previously described (Table 2, Fig. 2) (Frye *et al.*, 2006). Procedures for SDS-PAGE and immunoblotting have been described previously (Tønjum *et al.*, 1995, 1998).

### Pilus–DNA-binding assay in solution.

The DNA-binding assay was based on a method previously described by van Schaik *et al.* (2005), and used the same buffers and volumes. Briefly, microtitre plates were coated with poly-l-lysine, but not blocked with BSA (Sigma), and coated with salmon sperm DNA (200 μg ml^−1^). Blocking with 4 % (w/v) BSA was then performed. Pili, purified by the shearing and ammonium-sulfate precipitation method of Brinton *et al.* (1978), were biotinylated with sulfosuccinimidyl-6-(biotin-amido)hexonoate (Pierce), following the manufacturer's instructions, and allowed to bind for 2–3 h. Binding was detected with avidin–alkaline phosphatase (Sigma), using 4-nitrophenyl phosphate (Fluka) as substrate in developing buffer (50 mM Tris/HCl, 3 mM MgCl_2_, pH 9.6). Absorption was measured at 405 nm, with 490 nm as background. Purified DNA glycosylase MutY (Davidsen *et al.*, 2005) was used as a positive control for assessing DNA binding.

### DNA substrates.

The sequences of the oligonucleotide substrates, with and without DUSs, employed in DNA-binding assays are listed in Table 2. Salmon sperm DNA (Roche Diagnostics), *N. meningitidis* chromosomal DNA prepared in 0.01 M phosphate buffer (pH 7.4), as well as non-labelled oligonucleotides were used as competitive DNA.

### Labelling of DNA substrates.

Synthesized oligomers (3.5 pmol) (Table 2) were end-labelled with [*γ*-^32^P]ATP (GE Healthcare) or biotin (Invitrogen) using T4 polynucleotide kinase (New England Biolabs), as described by Sambrook *et al.* (1989). Double-stranded substrates were generated by mixing equal amounts of complementary oligomers, before heating to 95 °C for 5 min and slowly cooling to room temperature, which allowed the oligomers to hybridize. Labelled substrates were separated on 20 % non-denaturing PAGE and extracted by diffusion in water.

### Band-shift analysis.

For electromobility shift assays, 200 ng protein was mixed with 2 μl 5× gel shift buffer (250 mM MOPS, pH 7.5, 5 mM EDTA, 5 mM DTT, 25 mM MgCl_2_, 25 %, v/v, glycerol) in a final volume of 10 μl. Where indicated, competing DNA was added at 1–1400-fold excess 10 min prior to the addition of labelled substrate. Labelled DNA (2000 c.p.m.) substrate was added to the sample on ice, and the mixture was incubated at room temperature for 20 min. Electrophoresis was carried out on 6 % polyacrylamide gels in Tris/glycine/EDTA buffer. Gels were dried, exposed to a PhosphorImager cassette, and scanned in a PhosphorImager 445 SI or Typhoon scanner (both from GE Healthcare).

### Southwestern analysis.

For the solid-phase overlay assays, protein samples were separated by SDS-PAGE and transferred to nitrocellulose membranes. The membranes were equilibrated and incubated overnight in renaturing buffer (0.5 % BSA, 0.25 % gelatin, 0.2 % Triton X-100, 10 mM Tris/HCl, pH 7.5, 5 mM *β*-mercaptoethanol, 100 mM NaCl). Probing with biotinylated DNA substrates (Invitrogen) (Table 2) was done overnight in renaturing buffer. The membranes were washed three times with washing buffer (10 mM Tris/HCl, pH 7.5, 100 mM NaCl) before incubation with alkaline phosphatase-conjugated streptavidin (Chemicon), dilution in renaturing buffer, and subsequent detection using 5-bromo-4-chloro-3-indolyl phosphate (BCIP) and nitroblue tetrazolium (NBT) as substrates (Life Technologies).

### Electron microscope imaging of PilQ bound to biotin-labelled DNA.

PilQ complex was purified from *N. meningitidis* strain M1080 membranes as previously described (Collins *et al*., 2001, 2003). Ten-base-pair oligonucleotides for both strands corresponding to the DUS (5′-GCCGTCTGAA-3′) and a DUS-negative sequence with five residues changed by 5′ biotinylation (5′-GAAGTACGAC-3′) were purchased from MWG. For gold-labelling experiments, 10 μl streptavidin with 5 nm gold label (Sigma) was mixed with equimolar amounts of biotin-labelled DNA and incubated on ice for 12 h. These DNA–gold conjugates were then added to 10 μl purified PilQ at a protein concentration of 50 μg ml^−1^, and incubated for 24 h at 4 °C with gentle agitation. Samples were then centrifuged at 13 000 r.p.m. in a bench-top centrifuge for 5 min, before the supernatant was extracted and prepared for negative staining. Aliquots of the PilQ oligomer–DNA incubation mixture were adsorbed to freshly glow-discharged carbon-coated copper grids, prior to application of negative stain (4 %, w/v, uranyl acetate) for 30 s, and briefly blotted onto double-layered Whatman 50 filter paper. Grids were analysed using a Philips CM100 transmission electron microscope (Philips Electron Optics) operating at an accelerating voltage of 100 keV.

## RESULTS

### Purified pili from *N. meningitidis* and *P. aeruginosa* exhibit weak binding of DNA

**Fig. 1. f1:**
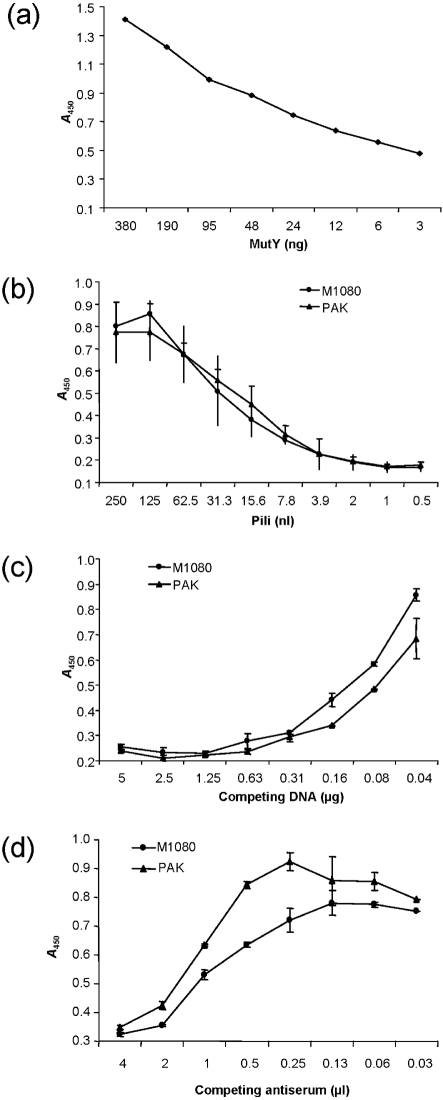
Detection of pilus–DNA interaction in solution. DNA was immobilized in the well and overlaid with protein in solution. (a) Binding of DNA glycosylase MutY to DNA (positive control protein). Saturation was reached at 1 μg MutY. One representative experiment out of four is shown. (b) Binding of *N. meningitidis* M1080 and *P. aeruginosa* PAK pili to DNA. The mean of four independent measurements using each purified pilus preparation is shown. Error bars indicate sd. (c) Competition of pili binding to immobilized DNA by preincubation with DNA in solution. The average of two independent measurements using pilus protein is shown, as above. Error bars indicate minimum and maximum values. (d) Competition of pili binding to immobilized DNA by preincubation with pilus-specific antibodies. The average of two independent measurements using 0.125 μl pilus proteins is shown, as above. Error bars indicate minimum and maximum values.

Interactions between purified *N. meningitidis* and *P. aeruginosa* pili and DNA were assessed in solution (Fig. 1). Various concentrations of protein and DNA were tested for binding under several different conditions, and the protein–DNA interaction was monitored using DNA glycosylase MutY as a positive control (Fig. 1a[Fig f1]). This analysis showed that assembled *N. meningitidis* and *P. aeruginosa* fimbrial structures weakly bound DNA under the conditions employed (Fig. 1b[Fig f1]), and that binding could be inhibited by competing DNA- and pilus-specific antibodies (Fig. 1c, d[Fig f1]). Purified *N. meningitidis* and *P. aeruginosa* pili did not bind DNA in a band-shift assay (data not shown).

### Distribution of positively charged sequences within PilQ

**Fig. 2. f2:**
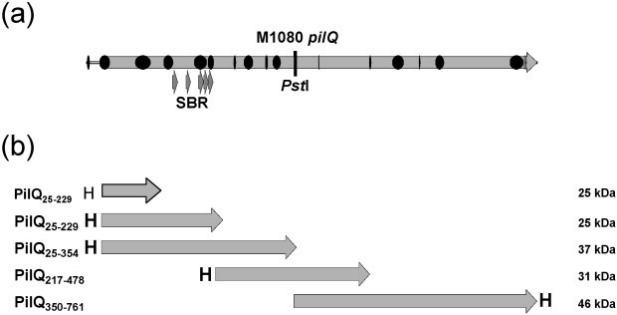
Gene organization and *pilQ* constructs. (a) Schematic representation of the *N. meningitidis* strain M1080 *pilQ* gene and the predicted gene product. The positions of the N-terminal SBRs and the *Pst*I restriction site are indicated. The locations of positively charged areas within the predicted sequence are marked in black. (b) Schematic representation of the PilQ truncated proteins used in this study, depicted relative to Fig. 2(a). The position of the hexa-histidine tag is indicated with an ‘H’ and the sizes of the recombinant proteins are given on the right.

The weak affinity of pili for DNA suggested that other pilus biogenesis components could contribute to DNA binding. The search for DNA-binding signatures in the predicted PilQ secondary structure, using various algorithms, did not reveal any recognized DNA-binding motifs such as helix–turn–helix, helix–loop–helix, leucine zipper or zinc finger motifs (data not shown). An analysis of the distribution of positively charged residues within the PilQ sequence showed that several stretches of such sequences were distributed throughout the reading frame (Fig. 2a[Fig f2]). In common with many DNA-binding proteins, some of these regions could be involved in mediating nucleic acid recognition (Misra & Honig, 1995). The N-terminal octameric repeat region (SBR) (Tønjum *et al.*, 1998) was one of the major positively charged regions identified.

### Observation of DNA binding to native PilQ complex using a band-shift assay

**Fig. 3. f3:**
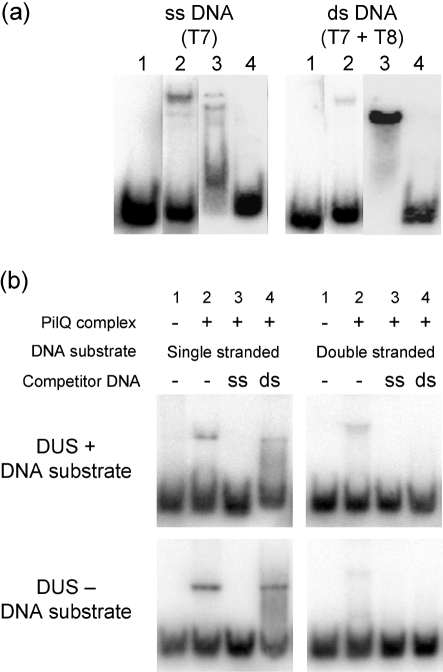
DNA-binding characteristics of the native PilQ complex measured by band-shift analysis. The interaction of native PilQ complex with ssDNA and dsDNA substrates was studied. (a) Band-shift analysis of PilQ complex binding to ssDNA (T7) or dsDNA (T7+T8 hybrid) substrates. Lane 1, free labelled DNA substrates (no PilQ); lane 2, DNA and PilQ complex; lane 3, DNA and *Taq* polymerase (positive control); lane 4, DNA and BSA (negative control). (b) Competition band-shift experiments, showing the ability of unlabelled ss- or dsDNA ligands to compete with the binding of labelled ligands. Results are compared for oligonucleotides containing (T1/T2) or not containing (T3/T4) DUSs. Lane 1, free labelled DNA substrates; lanes 2–4, PilQ complex.

**Fig. 4. f4:**
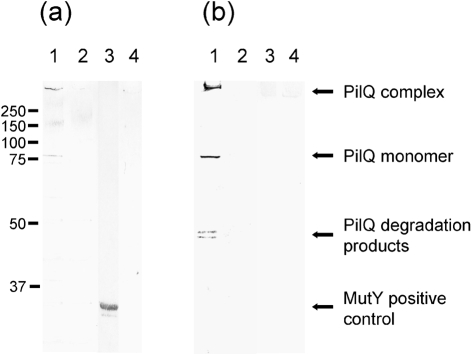
Binding of the PilQ complex to DNA assessed by a solid-phase overlay assay in the form of Southwestern analysis. Southwestern analysis of the proteins reacted with ssDNA substrate (T1) (a) and immunoblotting with PilQ-specific antibody (b) is shown. Lanes 1, M1080 whole-cell lysate; lanes 2, M1080-ΔPilQ whole-cell lysate; lanes 3, MutY (positive control); lanes 4, BSA (negative control). Markers on the left give the molecular mass in kDa; arrows on the right indicate the migration positions of the PilQ complex, PilQ monomer, PilQ degradation product typical for secretins, and DNA glycosylase MutY (positive control protein for DNA binding).

The PilQ complex, purified directly from meningococcal outer membranes, was assessed for DNA binding using band-shift assays. PilQ was mixed with radioactively labelled DNA substrates, some with and some without the DUS recognition sequence. The native PilQ complex showed a preference for binding to ssDNA, although some binding to dsDNA was also discernable (Fig. 3a[Fig f3]). In a competition assay, ssDNA was more effective at competing for binding than dsDNA (Fig. 3b[Fig f3]). To evaluate the possibility that DNA binding is dependent on the presence of the DUS or base composition, short oligonucleotides with various DUS contents were employed (Table 2). No sequence or DUS specificity in PilQ complex-mediated DNA binding was observed (Fig. 3b[Fig f3]). To validate the DNA-binding properties of PilQ, a solid-phase overlay assay in the form of Southwestern analysis was also employed (Fig. 4). The binding of ssDNA to the PilQ multimer, which runs at the top of the stacking gel, was readily apparent using samples from whole-cell lysates (Fig. 4a[Fig f4]). Similar results were obtained using dsDNA (data not shown), although binding of ssDNA was observed to be of higher affinity than that of dsDNA, irrespective of the presence or absence of DUS in the DNA substrate. The PilQ degradation product, routinely observed in secretin preparations and representing the C-terminal part of the monomer, did not bind DNA. This indicates that the C-terminal part of the PilQ monomer, which makes up the degradation product (Tønjum *et al.*, 1998), is not the region of PilQ that promotes binding of DNA.

### Investigation of DNA binding using recombinant PilQ fragments

**Fig. 5. f5:**
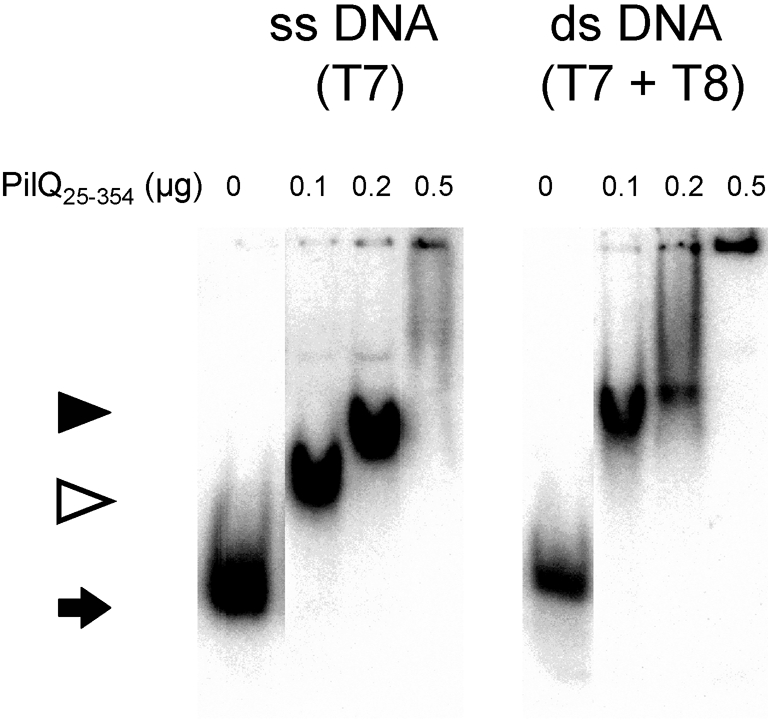
Interaction of recombinant PilQ with DNA substrates in band-shift analysis: dependence on protein concentration and DNA substrate. Free labelled (2000 c.p.m.) ssDNA T7 and dsDNA (T7+T8 hybrid) ligands were incubated with the indicated amount of recombinant N-terminal PilQ protein PilQ_25–354_, and run under standard conditions. The migration of the shifted DNA substrates appeared to be affected by the PilQ protein concentration and/or charge. Filled arrow, free oligonucleotide; open arrow, partial shift, typical of PilQ_25–354_; filled arrowhead, full band shift. At high protein concentrations, the sticking of oligonucleotides to protein in the well was also observed.

The results obtained in the band-shift and Southwestern assays established that native PilQ, purified directly from meningococcal membranes in an assembled state, was able to bind various DNA oligomers. When optimizing the protein–DNA ratio, it was found that 200 ng protein was adequate, which is the amount of protein regularly employed in band-shift assessments of, for example, DNA repair proteins (Davidsen *et al.*, 2005). In order to determine the PilQ region responsible for mediating DNA binding, a range of recombinant fragments of PilQ was prepared (Fig. 2b[Fig f2]). ssDNA- and dsDNA-binding capability was detected for some of the recombinant PilQ fragments (Fig. 5, Table 4). The PilQ_25–354_ fragment exhibited the most evident DNA-binding capability in band-shift analysis. Interestingly, increasing protein concentrations produced increasing band-shift patterns for ssDNA, as well as for dsDNA (Fig. 5). The partial band shifts observed might have been due to protein degradation during non-saturated binding of DNA, in combination with the effect of the electrostatic charge of this protein on its migration pattern.

The determination of the binding of all four recombinant PilQ fragments and control proteins to ssDNA and dsDNA by band-shift analysis is shown in Table 3. Among the PilQ recombinants, the PilQ_25–354_ protein bound DNA much more strongly than did the other proteins, mapping the region mediating DNA binding to N-terminal PilQ, and corroborating the results from the solid-phase overlay analysis (Fig. 4). Further testing of chimeric meningococcal PilQ complex from strain TT31-221, in which the N-terminal positively charged SBR region is replaced by the corresponding but non-homologous region in *P. aeruginosa* PilQ (Tønjum *et al.*, 1998), demonstrated that this macromolecule exhibited reduced DNA binding only (data not shown). As described above, the wild-type PilQ complex bound more readily to ssDNA than to dsDNA, and dsDNA was less competitive than ssDNA. One exception to this finding was the N-terminal PilQ_25–354_ fragment, which bound both dsDNA and ssDNA (Table 3, Fig. 5). Analysis of other recombinant truncated PilQ proteins showed that they and the neisserial outer-membrane proteins (HmbR and OpcA) employed as negative controls did not bind DNA (Table 3). Competitive band-shift analysis with the N-terminal PilQ_25–354_ fragment confirmed that both dsDNA and ssDNA compete for binding to this particular protein, although ssDNA was a more effective competitor than dsDNA (Table 4). The more potent effect of ssDNA in competition for DNA-binding affinity was verified by adding a surplus of competing non-labelled salmon sperm or meningococcal chromosomal DNA (data not shown).

### Examination of PilQ complex–DNA interaction by electron microscopy

**Fig. 6. f6:**
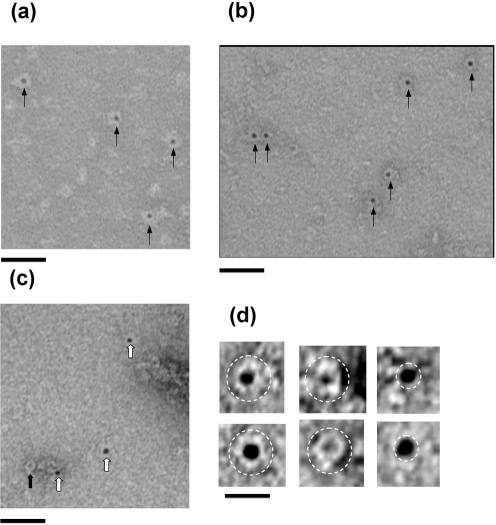
Interaction between DNA and the PilQ complex visualized by transmission electron microscopy. (a) PilQ complex incubated with avidin–nanogold plus biotinylated dsDNA; complexes are indicated with arrows. Bar, 50 nm. (b) PilQ complex incubated with avidin–nanogold plus biotinylated ssDNA; complexes are indicated with arrows. Bar, 50 nm. (c) PilQ complex incubated with avidin–nanogold; nanogold particles alone are indicated by white arrows, and an unliganded PilQ particle is indicated by the black arrow (negative control). Bar, 50 nm. (d) Panel of example particles in each class: left column, PilQ–avidin–nanogold plus biotinylated ssDNA; middle column, unlabelled PilQ; right column, avidin–nanogold label alone. The perimeter of each particle is indicated. Bar, 16.5 nm. For clarity, the contrast in all the figures has been enhanced.

The binding of DNA to the PilQ complex was studied by transmission electron microscopy. Biotinylated DNA was incubated with the native PilQ complex, and binding detected with an avidin–nanogold conjugate (Fig. 6). Control incubation, which contained just PilQ and the avidin–nanogold reagent, showed evidence for separate PilQ (Fig. 6c[Fig f6], black arrow) and nanogold particles (white arrows). Pre-incubation of the avidin–gold with either ds- or ss-biotinylated DNA resulted in labelling of the PilQ complex in a highly specific manner (Fig. 6a, b[Fig f6], arrows). This was apparent from the stain exclusion visible around the circumference of the nanogold particles (Fig. 6d[Fig f6], compare left with right column). High electron density was consistently found at the centre of the projection of the complex, rather than at the periphery. The results suggested that DNA binding to PilQ is mediated by a specific site or sites within the PilQ complex.

## DISCUSSION

*N. meningitidis* is naturally and constitutively competent for transformation, and the process is coupled to the expression of type IV pili. However, the exact relationship between the transformation process, pili and pilus biogenesis components is poorly understood. We asked whether neisserial pili or PilQ binds DNA, facilitating the entry of DNA during transformation. Therefore, we investigated the ability of these components to bind DNA by band-shift analysis, in solution and Southwestern analysis, as well as by electron microscopy. We demonstrated that neisserial and *P. aeruginosa* type IV pili bind DNA only weakly in solution. However, recombinant PilQ monomers and native PilQ complex purified from meningococcal outer membranes bound DNA from several sources. PilQ-mediated DNA binding was not DUS or sequence specific. Interestingly, binding of ssDNA to PilQ was preferred over the binding of dsDNA. The binding appeared to take place in the centre of the PilQ ring-like projection, rather than at the periphery, suggesting that the DNA-binding sites are located within the central portions of the PilQ oligomer. The band-shift analysis of truncated recombinant PilQ monomers showed that the N-terminal PilQ_25–354_ was the region predominantly involved in DNA binding. Analysis of the PilQ complex made up of chimeric subunits lacking the N-terminal SBR region exhibited much-reduced DNA binding, compared to that of the wild-type PilQ complex. We have previously shown that N-terminal PilQ is predominantly located in the periplasm (Frye *et al.*, 2006), and most likely also contributes to forming the central channel of the PilQ complex. Electrostatic charge prediction of the deduced PilQ amino acid sequence suggested that there is a region of increased positive charge in the N-terminal portion of this secretin (Fig. 2a[Fig f2]).

Electron microscopy studies have shown that secretins form stable doughnut-like structures in projection, with the diameter of the central cavity ranging from 6 to 8.8 nm (Bitter *et al.*, 1998; Collins *et al.*, 2003; Nouwen *et al.*, 2000). Electrophysiological measurements have shown that secretins can indeed form aqueous channels (Nouwen *et al.*, 1999). Clearly, such large channels must be gated to preserve the integrity of the outer membrane and periplasmic compartment. Among these secretins, the best characterized is PilQ from *N. meningitidis*, which also functions in pilus biogenesis (Tønjum *et al.*, 1998). The diameter of the central cavity in the PilQ 12-mer (Collins *et al.*, 2003) fits the proposed pilus fibre model (∼6 nm diameter) (Collins *et al.*, 2003, 2004), and could therefore easily accommodate the DNA double helix (∼2.4 nm). DNA uptake in *N. meningitidis* requires the presence of a DUS, although no DUS specificity in PilQ DNA binding was observed. This suggests that DUS specificity is conferred at another level. The conundrum that competence for transformation is dependent on pilus expression precludes some of the biological testing one would like to perform to validate the biological significance of the DNA-binding properties detected.

The binding and uptake of transforming DNA into the meningococcal cell can be divided into four stages: entry through the outer membrane, transit of the periplasm, transport across the inner membrane, and integration of the new DNA into the chromosome. We propose that the early part of meningococcal transformation is coupled to pilus retraction, and that transforming DNA is introduced into the cell through the transiently opened PilQ channel in the wake of the retracting pili. A similar model has been suggested for *P. stutzeri* (Graupner *et al.*, 2001). We further suggest that DNA is introduced to the inner membrane through the positively charged channel formed by the PilQ complex, and that other DNA-binding components exert sequence specificity and process DNA. In such a scenario, ComP (as a composite of the pilus structure or as a separate structure) (Aas *et al.*, 2002) could contribute by catching DNA and presenting it to DNA-binding components such as PilQ and ComE (Chen & Gotschlich, 2001). During these events, one strand of the DNA must be degraded and the ssDNA transported through the inner membrane. The presence of ssDNA in the cytoplasm and periplasm has been detected by Hill and co-workers (Chaussee & Hill, 1998). Degradation of one strand of the transforming DNA has until now been suggested to take place in the periplasm (Chaussee & Hill, 1998), although no recognized nuclease has so far been found. The fact that ssDNA binding to PilQ predominates over dsDNA binding indicates that during transformation either one strand of the transforming DNA is degraded or the DNA-binding epitopes of PilQ are located at the periplasmic surface of the outer membrane, which agrees with recent data on PilQ topology (Frye *et al.*, 2006). On the other hand, there might be a substantial amount of ssDNA in the natural environment of *N. meningitidis* at the mucosal surface. Wackernagel and co-workers have demonstrated that *P. stutzeri* can be transformed by ssDNA (Meier *et al.*, 2002), and an abundance of ssDNA has been demonstrated in the *N. gonorrhoeae* periplasm during transformation (Chaussee & Hill, 1998). Yet another explanation might be that the PilQ-mediated binding of ssDNA is attributable to functions other than transformation, such as phage transduction and/or conjugation. PilQ has recently been described as the secretin used by a filamentous phage (Bille *et al.*, 2005). The genomes of these phages are ssDNA, and the DNA has to interact with PilQ from the periplasm. In addition, no DUS is required for phage production or conjugation.

No pilus–DNA interaction could be detected by band-shift analysis, in either *N. meningitidis* or *P. aeruginosa*, probably due to steric hindrance. The lack of DNA binding by neisserial pili in a solid-phase overlay has been demonstrated by Mathis and Scocca (1984). We could, however, detect weak *N. meningitidis* and *P. aeruginosa* pilus-mediated DNA binding when employing a solution-based assay, corroborating the findings of van Schaik *et al.* (2005) for *P. aeruginosa* pili. An extended positively charged surface patch has been proposed for *P. aeruginosa* pili, and this patch is suggested to be responsible for binding DNA without sequence specificity (van Schaik *et al.*, 2005). Tainer and co-workers have, based on their structural analysis, predicted similar positively charged patches or grooves along the assembled neisserial pilus (Parge *et al.*, 1995). If electrostatic charge is important in promoting protein–DNA interactions, the neisserial pilin subunit PilE (theoretical pI 9.19) should be even more prone to charge-mediated interaction with the negatively charged DNA than the *P. aeruginosa* pilin subunit PilA (theoretical pI 6.24). Furthermore, it has been demonstrated that the *pilA* gene of *P. aeruginosa* can complement a *pilE* null mutant in *N. gonorrhoeae* to regain competence, implying that, in neisserial transformation, the DUS specificity is not imparted by the neisserial pilus subunit (Graupner *et al.*, 2001). Based on these collective findings, we propose that binding of neisserial and *P. aeruginosa* pili to DNA takes place only to a minor extent compared to the DNA binding exerted by PilQ.

We believe that we have described for the first time that the outer-membrane protein PilQ binds DNA in a non-DUS-specific manner. Based on these findings, we suggest that the PilQ complex is involved in non-sequence-specific and DUS-independent DNA binding in the meningococcus during the transformation process. The ability of the PilQ complex, and to some extent pili, of *N. meningitidis* to bind DNA could therefore contribute to the presentation of DNA to the meningococcal cell during the early part of natural transformation. In addition, the interactions among macromolecules such as PilQ, type IV pili and DNA could be involved in biofilm formation during mucosal surface colonization, or in the course of infection. The ultimate goal is to define how DNA-binding components are involved in the dynamic multi-site targeting, entry and processing of DNA during natural transformation.

## Figures and Tables

**Table 1. t1:** Primers used in this study Restriction sites are underlined.

**Primer**	**Orientation in relation to *pilQ***	**Sequence (5′–3′)**	**Restriction site**
Q*Bam*HI	+	CGGGATCCGGAAACATTACAGACATCAAAG	*Bam*HI
PilQ4B	−	GCGGATCCGCATCAATAGCGCAGGCTGTTGC	*Bam*HI
SF20	+	GCCCATGGCCATCCTGCAGATTTTGGC	NcoI
SF19	−	GCCTCGAGATAGCGCAGGCTGTTGCCGG	*Xho*I
SF21	+	GCGGATCCACCAATATCGATTTCCGCAAAG	*Bam*HI
SF22	−	GCAAGCTTGGCGGGATCGATCAGCACGCTG	*Hin*dIII
SF13	+	ATCGGACGATACCGTGTCC	−
SF14	−	TCGATATTGGTTTGTTTTGCTG	−
SF7	+	GACCGTCAAAATCAACAAGGA	−
SF8	−	AATACCGCCGACAATCAATG	−
SF1-for	+	AAGAGGCCAAAATCGAATCC	−
SF1-rev	−	CAGGCGAGTCCTTGTTGATT	−
SF9	+	GCGAATTCCATCGCCTTGGACTTTGAAC	*Eco*RI
SF12b	−	GCGGATCCTGAGGGAGAGGGTCATTTTG	*Bam*HI
PilQ1	+	GCGAATTCGCGTGCTGATCGATCCCGCC	*Eco*RI
PilQ4c	−	GCGAATTCGCATCAATAGCGCAGGCTGTTGC	*Eco*RI

**Table 2. t2:** Oligonucleotide substrates used in this study SUD, DNA uptake sequence 3′–5′ (DUS is 5′–3′). DUS and SUD sequences are shown in bold type.

**Oligonucleotide**	**Sequence (5′–3′)**	**Pyrimidine content (%)**	**DUS**
T1	CAACAACAACAACA**GCCGTCTGAA**CCAAA**TTCAGACGGC**AACAACAACAACA	40	DUS+SUD
T2	TGTTGTTGTTGTT**GCCGTCTGAA**TTTGG**TTCAGACGGC**TGTTGTTGTTGTTG	60	DUS+SUD
T3	CAACAACAACAACA**G**G**C**C**T**G**T**C**A**TCCAAA**A**T**G**A**C**A**G**G**C**CAACAACAACAACA	40	None
T4	TGTTGTTGTTGTTG**G**C**C**T**G**T**C**A**T**TTTGGA**T**G**A**C**A**G**G**C**C**TGTTGTTGTTGTTG	60	None
T7	CCGCCGCCT**GCCGTCTGAA**AGATA**TTCAGACGGC**ATCGGC	52	DUS+SUD
T8	GCCGAT**GCCGTCTGAA**TATCT**TTCAGACGGC**AGGCGGCGG	48	DUS+SUD
T9	CAACAACAACAACA**GCCGTCTGAA**CCAAA**GCCGTCTGAA**AACAACAACAACA	40	DUS+DUS
T10	TGTTGTTGTTGTT**TTCAGACGGC**TTTGG**TTCAGACGGC**TGTTGTTGTTGTTG	60	SUD+SUD
T11	CAACAACAACAACACTGTCAGTTGCCAAAGACAGTCAACAACAACAACAACA	40	None
T12	GTTGTTGTTGTTGTGACAGTCAACGGTTTCTGTCAGTTGTTGTTGTTGTTGT	60	None

**Table 3. t3:** Interaction of recombinant truncated PilQ proteins in band-shift analysis with ss- and dsDNA ssDNA comprised primers T1, T2, T3, T7 and T8, and dsDNA comprised T1+T2, T3+T4 and T7+T8 hybrids (see Table 2). +, Positive reactivity in the band shift; (+), weak binding of DNA; −, no visible band shift.

**PilQ recombinant**	**DNA substrates**								
	**ssDNA**						**dsDNA**		
	**T1**	**T2**	**T3**	**T4**	**T7**	**T8**	**T1+T2 hybrid**	**T3+T4 hybrid**	**T7+T8 hybrid**
	**DUS+**	**DUS+**	**DUS−**	**DUS−**	**DUS+**	**DUS+**	**DUS+**	**DUS−**	**DUS+**
PilQ_25–132_	−	−	−	−	−	(+)	(+)	+	(+)
PilQ_25–354_	+	+	+	+	+	+	+	+	+
PilQ_218–478_	−	(+)	−	−	(+)	−	−	(+)	(+)
PilQ_352–761_	−	−	−	−	−	−	−	−	−
OpcA*	−	−	−	−	−	−	−	−	−
HmbR*	−	−	−	−	−	−	−	−	−
MutY†	(+)	(+)	(+)	(+)	(+)	(+)	+	+	+

*Negative control. †Positive control.

**Table 4. t4:** Competitive DNA band-shift analysis with recombinant N-terminal PilQ The interaction of recombinant N-terminal PilQ_25–354_ with DNA with and without the DUS is shown. DNA substrates were single stranded (T1, T3) and double stranded (T1+T2 hybrid, T3+T4 hybrid). Competition with non-labelled competitor ss- or dsDNA is indicated. +, Reactivity in the band shift; (+), weak binding of DNA; −, no visible band shift.

**DNA substrate**	**Competing DNA**		
	**None**	**ssDNA**	**dsDNA**
T1/DUS+	++	−	+
T1+T2/DUS+	++	−	−
T3/DUS−	++	−	(+)
T3+T4/DUS−	++	−	−

## Abbreviations

- DUS, DNA uptake sequence
- SBR, small basic repeat
